# Differences in demographic composition and in work, social, and functional limitations among the populations with unipolar depression and bipolar disorder: results from a nationally representative sample

**DOI:** 10.1186/1477-7525-9-90

**Published:** 2011-10-13

**Authors:** Nathan D Shippee, Nilay D Shah, Mark D Williams, James P Moriarty, Mark A Frye, Jeanette Y Ziegenfuss

**Affiliations:** 1 Knowledge and Evaluation Research Unit, Mayo Clinic, Rochester, Minnesota, USA; 2 Division of Health Care Policy and Research, Mayo Clinic, Rochester, Minnesota, USA; 3 Department of Psychiatry and Psychology, Mayo Clinic, Rochester, Minnesota, USA

## Abstract

**Background:**

Existing literature on mood disorders suggests that the demographic distribution of bipolar disorder may differ from that of unipolar depression, and also that bipolar disorder may be especially disruptive to personal functioning. Yet, few studies have directly compared the populations with unipolar depressive and bipolar disorders, whether in terms of demographic characteristics or personal limitations. Furthermore, studies have generally examined work-related costs, without fully investigating the extensive personal limitations associated with diagnoses of specific mood disorders. The purpose of the present study is to compare, at a national level, the demographic characteristics, work productivity, and personal limitations among individuals diagnosed with bipolar disorder versus those diagnosed with unipolar depressive disorders and no mood disorder.

**Methods:**

The Medical Expenditure Panel Survey 2004-2006, a nationally representative survey of the civilian, non-institutionalized U.S. population, was used to identify individuals diagnosed with bipolar disorder and unipolar depressive disorders based on ICD-9 classifications. Outcomes of interest were indirect costs, including work productivity and personal limitations.

**Results:**

Compared to those with depression and no mood disorder, higher proportions of the population with bipolar disorder were poor, living alone, and not married. Also, the bipolar disorder population had higher rates of unemployment and social, cognitive, work, and household limitations than the depressed population. In multivariate models, patients with bipolar disorder or depression were more likely to be unemployed, miss work, and have social, cognitive, physical, and household limitations than those with no mood disorder. Notably, findings indicated particularly high costs for bipolar disorder, even beyond depression, with especially large differences in odds ratios for non-employment (4.6 for bipolar disorder versus 1.9 for depression, with differences varying by gender), social limitations (5.17 versus 2.85), cognitive limitations (10.78 versus 3.97), and work limitations (6.71 versus 3.19).

**Conclusion:**

The bipolar disorder population is distinctly more vulnerable than the population with depressive disorder, with evidence of fewer personal resources, lower work productivity, and greater personal limitations. More systematic analysis of the availability and quality of care for patients with bipolar disorder is encouraged to identify effectively tailored treatment interventions and maximize cost containment.

## Introduction

Mood disorders are among the most prevalent and costly health problems in the U.S. These conditions--which include unipolar (major depression, dysthymia, depression NOS) and bipolar disorders (bipolar types I and II, bipolar NOS)--are not uncommon. In the U.S., the 12-month prevalence rate for any mood disorder is approximately 9.5% [1]. Furthermore, mood disorders incur a massive economic burden, including millions of dollars in direct costs, such as health care expenditures [2-5]. Total costs reach into the billions after adding indirect costs, such as diminished work productivity [6-10].

Mood disorders are neither identical nor uniformly distributed, and differ in their respective impacts. Bipolar disorder not only carries unique symptoms (e.g., mania/hypomania), but also is distinct from unipolar depression in its prevalence and costs. For instance, whereas the 12-month prevalence of major depression is approximately 6.7% [1], it is between only 2% and 2.6% for bipolar disorder I and II [1,11]. Also, there is some evidence that the population distribution of bipolar disorder differs *demographically *(by age, sex, etc.) from the populations with depression or with neither condition [12,13]. In addition, despite lower prevalence, the total economic costs are relatively higher for bipolar disorder than for depression [14,15]. In fact, compared to several other conditions, bipolar depression had the highest percentage of cost in relation to work absences or short term disability [16].

The costs of mood disorders and other conditions are not limited to health care or work productivity. For an affected individual, the impact of mood disorders is diffused throughout daily life via physical, cognitive, and social limitations, such as poorer psychomotor control, attention deficits, and disrupted social role functioning [17-19]. Here again, bipolar disorder may incur particularly high disablement due to greater numbers of depressive episodes [20], higher functional impairment [21], and more prominent cognitive impairment or psychoses [22,23]. Still, despite the potentially far-reaching implications of these limitations for the individual and society, they are more difficult to detect or quantify than work absenteeism or financial costs. Consequently, evidence regarding the individual (versus economic or societal) costs of mood disorders--and especially how these costs manifest among populations with bipolar disorder versus depression and no mood disorder--is extremely limited.

The unique prevalence and costs of bipolar disorder provide our point of departure. The U.S. population with bipolar disorder is a potentially unique and vulnerable group. Yet, despite a small amount of existing literature [21], the differences in prevalence and costs between populations with unipolar depressive disorders versus bipolar disorder remain unknown, hindering the potential for effectively targeting these populations with mental health programming and policy. Furthermore, analyses at the level of individuals impacted by mood disorders, especially concerning bipolar disorder, are largely absent. The goals of this study are 1) to assess the demographics of mood disorder populations at a national level, and 2) to measure the distinct societal and individual costs for patients with bipolar disorder versus patients those with depression or no mood disorder.

## Methods

This study was deemed exempt of Institutional Review Board (IRB) approval by the Mayo Clinic Rochester IRB.

### Data and study population

The Medical Expenditure Panel Survey (MEPS) 2004-2006 Household and Medical Condition files were used to identify individuals with mood disorders. The MEPS is an ongoing study conducted by the Agency for Healthcare Research and Quality (AHRQ) that began in 1996. A nationally representative survey of the U.S. civilian, non-institutional population, the MEPS is designed to collect information about health status, medical care use, and expenditures, along with demographic and socioeconomic characteristics of the population. It utilizes an overlapping panel design in which individuals are interviewed five times over a period of 30 months [24]; from this, annualized estimates of population characteristics, health, and health care can be produced [25]. Although the MEPS collects data about people of all ages, the focus of the current study was limited to those aged 18 to 64.

### Measures

Diagnoses of unipolar depression and bipolar disorders were based on the ICD-9 classification system. Detailed ICD-9 codes were obtained at the National Center for Health Statistics Research Data Center in Hyattsville, MD. Diagnoses of 296.00-296.16 or 296.40-296.99 in any wave of the MEPS panel were classified as bipolar disorder. Diagnoses of 296.20-296.36, 300.40 or 311 were classified as depression. Individuals with a diagnosis of bipolar disorder, with or without a diagnosis of depression, were classified as bipolar disorder. Individuals with a diagnosis of depression and no diagnosis of bipolar disorder were classified as depression. Remaining individuals comprised the non-mood disorder population, including all non-institutionalized U.S. adults without diagnoses of bipolar disorder or depression. No distinction was made within these three groups regarding diagnoses of alcohol disorders, schizophrenia or other psychotic disorders.

The key outcomes of interest pertain to the indirect costs of mood disorders, namely the lost work productivity and personal limitations associated with a diagnosis of bipolar disorder or a depressive disorder. Both types of costs can be thought of as morbidity or productivity costs, i.e., the "lost or impaired ability to work or engage in leisure time activities due to morbidity" [26]. Lost work productivity was the more conventional among cost of illness studies [27,28], and pertained to workforce participation and absenteeism. This was assessed with three related items. The first concerned whether individuals were employed (full- or part-time) or were full-time students. The second, for individuals who were employed, concerning whether an individual had missed at least 10 days of work (i.e., two work weeks) in a year due to illness. Third, to further assess the extent of lost productivity, we also employed an item regarding whether the individual had spent at least 10 days of missed work in bed. Personal limitations were more unique among extant literature, and concerned the impact of mood disorders on individual functioning and self-sufficiency. This was measured via self reports of: 1) physical limitations (defined as "difficulty in walking, climbing stairs, grasping objects, reaching overhead, lifting, bending or stooping, or standing for long periods"); 2) social limitations (on "participation in social, recreational, or family activities"); 3) cognitive functioning (confusion, memory loss, or problems in decision-making that interfered with daily activities); or 4) being "limited, in any way, in the ability to work at a job, do housework, or go to school." We recognize that distinctions between productivity and personal limitations are somewhat arbitrary, as personal functioning is certain to affect one's ability to work. The measures of lost productivity and self-reported limitations, moreover, are in some cases very similar. However, we do not claim that these domains are unrelated; rather, we use this approach in order to explore the pervasive disablement among the populations with bipolar disorder and depression.

Covariates of interest included gender; age; categories for race/ethnicity; marital status (married versus not married); income; education; living arrangement (living alone versus living with another adult and/or child); an individual count of comorbid conditions (out of 15 total conditions including myocardial infarct, cardiovascular disease, dementia, ulcers, liver or kidney disease, diabetes, AIDS, cancer, and others used in the Charlson comorbidity index [29]); geography (living in a metropolitan statistical area versus not); and region (living in the Northeast, Midwest, South, or Western U.S.).

### Analytic Approach

Due to the relatively small sample of individuals with bipolar disorder in MEPS, estimates from the 2004-2006 MEPS were combined, representing an annualized three-year average over this time period. All analyses employed survey weights to represent the U.S. adult, non-institutionalized population. The weights also accounted for panel attrition over the two years that individuals were in the MEPS. Analyses were performed using StataSE 10.0 in order to account for the complex survey design of the MEPS. All reported differences are significant at p < 0.05, unless otherwise noted.

We compared the population with bipolar disorder to those with depression and with no mood disorder, with respect to a) demographic composition, and b) work and personal impact. T-tests for independent samples served to detect significant differences between populations. To ensure that the findings from bivariate analyses were not driven by underlying demographic patterns, we used logistic regression to isolate the independent impacts of bipolar disorder and depression on work productivity and personal limitations. Multivariate analyses of work impact were also subdivided into full-sample and gender sub-sample analyses due to the potential for unemployment or missed work to be differentially distributed along gender lines.

## Results

Weighted estimates for the population indicated 1.65 million individuals with a diagnosis of bipolar disorder (0.9% of the adult population), and 16.9 million individuals with depression (9.2% of the adult population; see Table 1). Compared to the population with depressive disorders, the population diagnosed with bipolar disorder was generally younger, not married, poorer (especially in the lowest income category), more commonly living alone, and less educated (with a lower proportion holding at least a college degree). Compared to the non-mood disorder population, the bipolar disorder population was generally female, non-Hispanic white or multiple-race, not married, poorer (again concentrated in the lowest income category), less educated (again, a lower proportion holding at least a college degree), living alone or living with only a child more prevalently (and living with another adult, or an adult and a child, less prevalently), and less often free of comorbid conditions. Also, the bipolar disorder population tended to cluster more in the central age range (35-44), giving it a narrower age distribution than the non-mood disorder population.

**Table 1:** Prevalence of and characteristics within individuals with bipolar disorder or depression compared to the non-mood disorder population, adults 18-64, United States, 2004-2006

	Bipolar disorder	Depression		Non-mood disorder	
	(n = 572)	(n = 5,464)		(n = 53,905)	
Total U.S. Population (18-64)	1,647,408	16,874,994		165,702,423	
	0.9%	9.2%		90.0%	

**Gender**					

Male	35.2%	33.3%		51.1%	*
Female	64.8%	66.7%		48.9%	*

**Age**					

18-24	11.4%	9.7%		16.1%	*
25-34	20.7%	16.2%		22.0%	
35-44	30.3%	23.0%	*	23.1%	*
45-54	25.1%	28.8%		22.3%	
55-64	12.5%	22.3%	*	16.6%	*

**Race/Ethnicity**					

Hispanic	8.1%	9.4%		14.8%	*
NH White	74.3%	77.9%		65.8%	*
NH Black	9.2%	7.8%		12.3%	
NH Multiple	4.7%	2.4%		1.2%	*
NH Other	3.8%	2.5%		6.0%	

**Marital status**					

Married	35.8%	48.1%	*	56.3%	*
Other	64.2%	51.9%	*	43.8%	*

**Income (% Federal Poverty Level)**					

< 200%	39.0%	21.3%	*	13.5%	*
200-399%	39.3%	43.4%		43.4%	
> = 400%	21.7%	35.4%	*	43.1%	*

**Educational Attainment (24 and older)**					

Less than High School grad	16.6%	15.9%		14.3%	
High School grad	35.7%	31.9%		30.9%	
Some College	27.1%	24.7%		23.3%	
College degree or more	20.7%	27.4%	*	31.5%	*

**Living Arrangement**					

Living Alone	37.6%	25.6%	*	17.1%	*
Living with child and adult	24.5%	29.0%		40.3%	*
Living with adult only	29.4%	38.2%	*	38.4%	*
Living with child only	8.5%	7.2%		4.2%	*

**Comorbid conditions**					

0	78.8%	74.9%		88.3%	*
1	16.9%	19.5%		10.3%	*
2	2.9%	4.6%		1.2%	
3 or more	1.4%	1.1%		0.2%	

**Geography**					

Metropolitan Statistical Area (MSA)	19.9%	17.5%		16.1%	
Non-MSA	80.1%	82.5%		83.9%	

**Region**					

Northeast	18.8%	16.6%		18.7%	
Midwest	25.3%	25.6%		21.9%	
South	32.1%	33.9%		36.3%	
West	23.8%	23.9%		23.1%	

* Indicates statistical difference (p < .05) between the bipolar disorder population versus the depression population or between the bipolar disorder population versus the non-mood disorder population

Source: 2004-2006 MEPS

### Work Productivity

A significantly lower proportion of the bipolar disorder population was employed or enrolled as full-time students than in either the depressed or non-mood disorder populations (42.8% compared to 63.3% and 80.7%, respectively; see Table 2). Among those working, the bipolar disorder group had a higher average number of days missed, and a higher percentage of individuals who missed at least two weeks of work (22.5% versus 6.3%), than in the non-mood disorder population. Furthermore, a higher proportion of the bipolar disorder population reported spending at least two weeks of missed work in bed, compared to the depressed and non-mood disorder populations (14.9% versus 8.2% and 2.9%, respectively). In multivariate analyses for work/societal limitations, we subdivided the living arrangement variable into living with another adult, living with a child, or living with both (with living alone as the reference category)--rather than simply "living alone" versus "not living alone"--to ensure that children or single parenthood were not disproportionately responsible for missed work. Regardless, multivariate models (Table 3) echoed bivariate findings: compared with the non-mood disorder population, individuals with bipolar disorder had about 4.6 times the odds of not working (95% CI 3.52, 6.04), 3.56 times the odds of missing at least two weeks of work (95% CI 2.12, 6.04), and 4.6 times the odds of spending at least 10 missed work days in bed (95% CI 2.75, 7.80). In similar fashion, individuals with depression also had higher odds of work-related costs than those with no mood disorder, but their odds ratios (between 1.93 and 2.37) were consistently smaller than for individuals with bipolar disorder. Models separated by gender suggested that the societal/work impacts of both mood disorder categories were similar for men and women; the point estimates were in most cases higher for men, but the 95% confidence intervals for the genders (not shown) overlapped in all cases except depression's effect on not working.

**Table 2:** Self-reported societal limitations by individuals with bipolar disorder or depression compared to the non-mood disorder population, adults 18-64, United States, 2004-2006

	Bipolar disorder	Depression		Non-mood disorder	
**Employed/student status**					

Not working or not a student (if 18-23)	57.20%	36.70%	*	19.30%	*
Working or a student (if 18-23)	42.80%	63.30%		80.70%	*

**Missed days of work**					

Average	8.36	7.45		3.45	*

**Missed 2 weeks (10 days) or more of work**					

No, missed fewer	77.50%	85.20%		93.70%	*
Yes, missed 2 weeks or more	22.50%	14.80%		6.30%	*

**Missed days of work/spent in bed**					

Average	4.95	3.81		1.6	*

**Missed work and in bed 2 weeks (10 days) or more**					

No, missed fewer	85.10%	91.90%	*	97.10%	*
Yes, missed 2 weeks or more	14.90%	8.20%	*	2.90%	*

* Indicates statistical difference (p < .05) between the bipolar disorder population versus the depression population or between the bipolar disorder population versus the non-mood disorder population

Source: 2004-2006 MEPS

**Table 3:** Odds of self-reported societal limitations by mood disorder, from multivariate analyses

	Overall		Women		Men	
**Model outcome**	**OR**	**p-value**	**OR**	**p-value**	**OR**	**p-value**
	**(SE)**		**(SE)**		**(SE)**	

**Not working or not a student (if 18-23)**						

Bipolar disorder	4.61	< 0.001	3.99	< 0.001	7.48	< 0.001
	(0.63)		(0.62)		(1.71)	
Depression	1.93	< 0.001	1.72	< 0.001	2.65	< 0.001
	(0.10)		(0.11)		(0.23)	

**Missed 2 weeks (10 days) or more of work**						

Bipolar disorder	3.56	< 0.001	3.64	< 0.001	3.57	0.003
	(0.95)		(1.19)		(1.51)	
Depression	2.11	< 0.001	1.96	< 0.001	2.61	< 0.001
	(0.14)		(0.15)		(0.33)	

**Missed work and in bed 2 weeks (10 days) or more**						

Bipolar disorder	4.63	< 0.001	4.30	< 0.001	5.76	0.001
	(1.23)		(1.30)		(3.09)	
Depression	2.37	< 0.001	2.30	< 0.001	2.59	< 0.001
	(0.22)		(0.23)		(0.47)	

### Personal Limitations

Compared to both depression and no mood disorder, higher percentages of individuals diagnosed with bipolar disorder reported social, cognitive, household, and work functioning limitations (Table 4). Moreover, a greater proportion of the bipolar disorder population also had physical limitations than the non-mood disorder population. Any limitation in school, work, or household work was reported by 40% of individuals with bipolar disorder--a rate nearly 10 times that of the non-mood disorder population and double that of the depression population. In multivariate analyses (Table 5), bipolar disorder and depression were significant, positive predictors of each limitation, but odds ratios indicated more prominent disablement for bipolar disorder. While depression and bipolar disorder both had between 2.4 and 2.7 times the odds of physical limitations compared to no mood disorder, the differences were more notable among other limitations. For instance, depressed individuals had 2.9 times the odds of social limitations, relative to no mood disorder, but the odds ratio for bipolar disorder was 5.1. Cognitive limitations were especially striking: depression was associated with 3.9 times the odds of cognitive limitations--but bipolar disorder was associated with 10.8 times the odds of having cognitive limitations, relative to no mood disorder. Continuing this pattern, depression and bipolar disorder were associated with, respectively, 3.2 and 6.7 times the odds of work limitations, relative to no mood disorder. Finally, depression meant 2.7 times the odds of household limitations, whereas bipolar disorder meant 3.5 times the odds, relative to no mood disorder.

**Table 4:** Self-reported individual limitations by individuals with bipolar disorder or depression compared to the non-mood disorder population, adults 18-64, United States, 2004-2006

Limitation	Bipolar disorder	Depression		Non-mood disorder	
Physical Limitation	27.40%	22.80%		6.40%	*
Social Limitation	26.20%	14.00%	*	2.80%	*
Cognitive Limitation	31.20%	12.40%	*	1.90%	*
Work Limitation	39.20%	21.00%	*	4.60%	*
Household Limitation	21.20%	14.30%	*	2.90%	*
Any Limitation (work, household, school)	40.80%	22.00%		4.80%	

* Indicates statistical difference (p < .05) between the bipolar disorder population versus the depression population or between the bipolar disorder population versus the non-mood disorder population

Source: 2004-2006 MEPS

**Table 5:** Odds of self-reported individual limitations by mood disorder

Model Outcome	OR	SE	p-value
**Physical**			

Bipolar disorder	2.68	0.38	< 0.001
Depression	2.46	0.14	< 0.001

**Social**			

Bipolar disorder	5.17	0.78	< 0.001
Depression	2.85	0.20	< 0.001

**Cognitive**			

Bipolar disorder	10.78	1.82	< 0.001
Depression	3.97	0.30	< 0.001

**Work**			

Bipolar disorder	6.71	0.92	< 0.001
Depression	3.19	0.20	< 0.001

**Household**			

Bipolar disorder	3.47	0.65	< 0.001
Depression	2.71	0.19	< 0.001

## Discussion

Mood disorders carry large indirect costs in terms of lost productivity and personal burden. However, important differences exist between the populations identified as having bipolar disorder versus unipolar depression, in regards to demographics, work, and individual functioning. This translates into the bipolar disorder population having fewer resources, yet also greater disablement--i.e., it is a distinct, and particularly vulnerable, group.

In our analyses, the bipolar disorder population tended to be younger, poorer, less educated, and more often unmarried and living alone, than the population with unipolar depression (not to mention differences from the non-mood disorder population). These demographic differences suggest that those in the bipolar disorder population tend to have fewer resources and a more limited social safety net than the depression population. This has two implications. First, bipolar disorder does not merely represent a unique subset of affective and psychomotor symptoms [17,23]; rather, it also characterizes a population which is demographically different from the populations with depression and no mood disorder.

A second implication is that, due to the relative disadvantages among the bipolar disorder population vis-à-vis demographics and circumstances, individuals with bipolar disorder may often be particularly susceptible to the disruptive effects of mood disorders. This is especially problematic when one considers our findings regarding the high costs imposed by bipolar disorder. Namely, the bipolar disorder population had higher rates of non-employment, spending missed work days in bed, and limitations in social, cognitive, work, and household domains than in the depressed or non-mood disorder populations. Moreover, multivariate analyses revealed particularly high disablement for bipolar disorder (versus depression) in not being employed and in having social, cognitive, and work limitations. Our multivariate gender subgroup analysis indicated that neither gender is particularly safe from, or susceptible to, work limitations, even controlling for varying living situations, suggesting that mood disorders' impact on lost productivity endures across demographic and personal circumstances.

In sum, the bipolar disorder population is distinct from the depressed and non-mood disorder populations in its demographic characteristics and in the work costs and personal limitations it incurs. Individuals diagnosed with bipolar disorder face greater disablement, yet also have fewer social and financial resources to call upon in combating these limitations. Without specifically tailored intervention, the special vulnerability of this population may remain under-addressed, perpetuating the disproportionately high work costs and personal burden of bipolar disorder.

### Limitations

The present study has several limitations. First, approximately 38% of the bipolar disorder population also had a diagnosis of depression. No sensitivity analysis was performed to either exclude these individuals or categorize them within the depression population. We cannot say what kind of impact, if any, these individuals had on study results. Second, we do not know if the individuals in either mood disorder population were on any disability program. It is possible that those on disability programs would be more likely to report poor functioning if individuals believed that reporting good functioning could endanger disability benefits. Third, our outcome variables were based on self-reported responses of the individuals surveyed, rather than work/school records, more objective assessments of functioning, etc. No attempt is made in the MEPS to verify the responses for these items. Fourth, diagnoses of bipolar disorder and depression were based on individual responses and confirmed by administrative data, but were not confirmed by specific screening instruments or exams. As such, patients may be incorrectly categorized. Fifth, we do not include measurements of substance abuse disorders/alcoholism or other psychiatric disorders (e.g., schizophrenia) among our mood disorder or non-mood disorder populations. This limits our ability to further control or analyze the relationships between mood disorders and disablement. For instance, we do not examine whether alcohol plays a role in linking mood disorders to lost work or cognitive limitations; also, the non-mood disorder group could still have psychiatric visits for other issues. Finally, although we controlled for medical comorbidities, we did not explore them in detail in order to fully assess their impact on the relationship between mood disorders and work or personal costs.

## Conclusion

Individuals with mood disorders exhibited higher work costs and personal limitations than non-mood disorder population, and evidence indicated a particularly troubling combination of potentially lower resources and higher disablement associated with bipolar disorder. Addressing the particular vulnerability of patients with bipolar disorder is a necessity. Further empirical study and policy attention to the quality and availability of care for these patients may have a large societal payoff, by identifying effective interventions and strategies for containing the unique costs of bipolar disorder. For instance, it is vital that programs be designed to target the prominent personal limitations (especially cognitive and social) experienced by individuals with bipolar disorder. It is likely these limitations are partially responsible for the greater productivity costs found. By considering the broader impact of bipolar disorder in individuals' lives, a strong case is made to allocate resources toward the management of this disorder's extensive reach.

In addition, bipolar disorder carries high productivity costs, including unemployment and spending missed work time in bed. The patterns found here in the different measures for lost productivity suggest that measuring lost work time among only *employed *individuals is insufficient in detailing even the work costs of mood disorders. It is vital that studies include non-employed and non-student individuals in analyses, and also that they examine the fullest extent of lost productivity (i.e., what happens during missed work time--full incapacitation in bed or otherwise). It is possible that non-employment itself, stemming from cognitive, social, or other limitations, is the most excessive and least necessary economic cost of mood disorders. Furthermore, it is probable that spending time in bed (or in similar states of disengagement) during missed work may be especially detrimental to other health conditions, and may stimulate further negative mood, similar to rumination in unipolar depression or anxiety [30].

Finally, if our results indicate anything, it is that bipolar disorder represents not only a unique condition--one that is distinct from unipolar depression--but also a unique (and vulnerable) population. As such, relying on an umbrella category of "mood/affective disorders" may mask the differences between bipolar disorder and depression, and between the respective demographic groups who endure them. In turn, this lack of differentiation may obstruct effective policy or treatment. Tailoring policy decisions with consideration for the particular vulnerabilities of the bipolar disorder group is thus vital in optimizing effectiveness and attacking unnecessary costs. Successfully targeted mental health policy requires differentiation within mood disorders to account for the greater costs and vulnerability among the bipolar disorder population.

## List of Abbreviations

(IRB); Institutional Review Board; (MEPS): Medical Expenditure Panel Survey; (AHRQ): Agency for Healthcare Research and Quality.

## Competing interests

MF has grant support from Pfizer, National Alliance for Schizophrenia and Depression (NARSAD), National Institute of Mental Health (NIMH), National Institute of Alcohol Abuse and Alcoholism (NIAAA), and the Mayo Foundation. He is a consultant for Dainippon Sumittomo Pharma, Merck, and Sepracor. He has CME-supported activity for Astra-Zeneca, Bristol-Myers Squibb, Eli Lilly and Co., GlaxoSmithKline, Merck, Otsuka Pharmaceuticals, Pfizer, and Sanofi-Aventis. (No competing interests for speakers' bureau or financial interest/stock ownership/royalties).

(All other authors have no competing interests.)

## Authors' contributions

NShi contributed to conceptualization, drafting/revising the manuscript, supplementary analyses, and presentation of findings. NSha contributed to study conception, interpretation of results, and critical revisions of the manuscript. MW was involved in designing the study and drafting and revising the manuscript. MF contributed to study design, data collection strategy, and revising the paper in terms of presentation of findings and discussion. JM participated in drafting the manuscript, data collection, and statistical analyses. JZ participated in the design, completed analyses, and helped draft the manuscript. All authors read and approved the final manuscript.
